# Assessing the burden of medical impoverishment by cause: a systematic breakdown by disease in Ethiopia

**DOI:** 10.1186/s12916-016-0697-0

**Published:** 2016-10-21

**Authors:** Stéphane Verguet, Solomon Tessema Memirie, Ole Frithjof Norheim

**Affiliations:** 1 Department of Global Health and Population, Harvard T.H. Chan School of Public Health, 665 Huntington Avenue, Boston, MA 02115 USA; 2 Department of Global Public Health and Primary Care, University of Bergen, Bergen, Norway

**Keywords:** Medical impoverishment, Catastrophic health expenditure, Out-of-pocket costs, Direct medical payments, Financial risk protection, Poverty, Ethiopia

## Abstract

**Background:**

Out-of-pocket (OOP) medical expenses often lead to catastrophic expenditure and impoverishment in low- and middle-income countries. Yet, there has been no systematic examination of which specific diseases and conditions (e.g., tuberculosis, cardiovascular disease) drive medical impoverishment, defined as OOP direct medical costs pushing households into poverty.

**Methods:**

We used a cost and epidemiological model to propose an assessment of the burden of medical impoverishment in Ethiopia, i.e., the number of households crossing a poverty line due to excessive OOP direct medical expenses. We utilized disease-specific mortality estimates from the Global Burden of Disease study, epidemiological and cost inputs from surveys, and secondary data from the literature to produce a count of poverty cases due to OOP direct medical costs per specific condition.

**Results:**

In Ethiopia, in 2013, and among 20 leading causes of mortality, we estimated the burden of impoverishment due to OOP direct medical costs to be of about 350,000 poverty cases. The top three causes of medical impoverishment were diarrhea, lower respiratory infections, and road injury, accounting for 75 % of all poverty cases.

**Conclusions:**

We present a preliminary attempt for the estimation of the burden of medical impoverishment by cause for high mortality conditions. In Ethiopia, medical impoverishment was notably associated with illness occurrence and health services utilization. Although currently used estimates are sensitive to health services utilization, a systematic breakdown of impoverishment due to OOP direct medical costs by cause can provide important information for the promotion of financial risk protection and equity, and subsequent design of health policies toward universal health coverage, reduction of direct OOP payments, and poverty alleviation.

**Electronic supplementary material:**

The online version of this article (doi:10.1186/s12916-016-0697-0) contains supplementary material, which is available to authorized users.

## Background

High out-of-pocket (OOP) medical expenses can lead to impoverishment in many low- and middle-income countries [1, 2]. Often, individuals and households resort to a variety of strategies to cope with illness-related bills such as borrowing money from peers or selling assets [3]. Without pre-existing financing arrangements, like social insurance, household medical expenditure can often be categorized as “catastrophic” – defined as exceeding a certain fraction (e.g., 10 %) of total household expenditure [2, 4].

Well-designed health policies are critical for the minimization of financial risks tied to OOP direct medical expenses. In this respect, the World Health Organization’s 2010 World Health Report [5] argued for pathways to universal health coverage (UHC) accounting for the reduction of OOP direct payments and financial risk protection as one essential dimension to measure progress on the way to UHC. Likewise, the Sustainable Development Goal for health (SDG3) included a sub-target on achieving “universal health coverage, including financial risk protection and access to quality essential health services” [6]. The prevention of medical impoverishment, defined here as OOP direct medical costs pushing households below the poverty line or leading to catastrophic medical expenditure, is a major objective of health systems and policy instruments such as insurance and public finance [7]. Therefore, analyzing the composition of OOP direct medical payments per cause can help design health systems better at preventing such catastrophic expenditure, which have been estimated to be substantial [8].

In the majority of low- and middle-income countries, there is not necessarily a clear evidence base for the interventions to be covered by public finance [7, 9]. In particular, there has been no systematic examination of which specific diseases and conditions (e.g., tuberculosis, heart disease) drive OOP direct medical costs and subsequent impoverishment. Such a systematic examination could inform the design of an essential health benefits package.

Understanding the underlying composition of medical impoverishment and its disaggregation per condition can prove useful for several reasons. First, it can point to the links between disease and poverty and be used to examine the poverty consequences of epidemiological changes and guide the implementation of tailored poverty-focused policies. In this case, assessing illness-related poverty would account for all the financial implications of disease, including OOP direct medical costs (e.g., medications and consultation fees), transportation costs, and indirect costs (e.g., time and income losses); this is not the intent of this paper. Second, the disaggregation of medical impoverishment, defined as OOP direct medical costs pushing households under the poverty line, can help within the health sector in the design of appropriate policy solutions which provide high financial protection [10]. This approach can highlight the interventions to be financed by the health sector that can prevent catastrophic medical expenditure. Thus, this paper deliberately focuses on OOP direct medical payments only, as it is one essential dimension of UHC [5].

We propose a preliminary analytical approach to systematically assess the burden of impoverishment due to OOP direct medical costs per condition in a given country. We intend to expose, with simplicity, a framework which we subsequently apply to estimate medical impoverishment by cause in one particular country, as an example, Ethiopia.

## Methods

### Approach

We built on an existing cost and epidemiological model [11] to assess medical impoverishment, defined as OOP direct medical costs (e.g., consultation fees, payments for medications) pushing households below a given poverty line per condition in a given country.

The model incorporated a range of parameters, including disease-specific mortality estimates (e.g., per cause of death), case fatality ratios, patient OOP direct medical costs, health services utilizations (e.g., healthcare seeking behavior, such as the probability that a person seeks care for a specific illness conditional on having that illness), and wealth inputs (e.g., household income). The model estimated the OOP direct medical costs related to a specific condition at the population level. Subsequently, it calculated the corresponding number of cases of poverty that could be attributed to OOP direct medical costs tied to that specific condition. The quantification of those medical poverty cases relied on counting the number of households crossing a defined poverty line (e.g., country poverty line) due to condition-related OOP direct medical costs.

We applied the model to the case study of Ethiopia. First, for simplicity, we selected 20 conditions established as major causes of mortality in Ethiopia (whose estimates were immediately available from the Global Burden of Disease study [12]), and hence deemed to likely incur substantial OOP direct medical costs. For each condition, we obtained estimated mortality, from which we derived the number of cases using a case fatality ratio (the number of cases would equal the number of disease-related deaths divided by disease-specific case fatality ratio); second, we estimated the associated OOP direct medical costs. These costs included the direct medical costs only – there was no accounting of transport costs or time losses for the affected individuals, as our study focused on catastrophic medical costs only. In addition, we accounted for health services utilization, the percentage of individuals seeking care for a specific illness conditional on having that illness, and the combination of OOP direct medical costs and utilization allowed the estimation of incurred OOP direct medical costs. Finally, using the inputs described above, supplemented by a distribution of income in the Ethiopian population, we derived the total number of poverty cases due to OOP direct medical payments that could be attributed to each of the 20 conditions in Ethiopia.

### Input data

First, we used causes of death data as provided by the Global Burden of Disease study [12] systematic estimates for Ethiopia for the year 2013. This data was used for three reasons: (1) the critical importance of leading causes of mortality in global health priorities; (2) its simplicity and tractability by restriction to 20 well-known causes constituting a large share of total mortality (70 % of the total deaths estimated in Ethiopia for 2013); and (3) its immediate availability. As a comparator, no data on disease-specific cases, incidence, or morbidity was immediately available in a systematic way per condition as was available for the causes of death data used herein. The causes of death included (1) communicable, maternal, and perinatal causes (neonatal conditions, respiratory infections, HIV/AIDS, diarrhea, tuberculosis (TB), malaria, meningitis, childhood vaccine-preventable diseases); (2) non-communicable diseases (vascular or diabetic, chronic respiratory, cirrhotic, cancers); and (3) injuries (road traffic accidents). The 20 causes of death captured approximately 70 % of the total deaths estimated to have occurred in Ethiopia in 2013 [12]. Subsequently, for each cause of death, the number of corresponding disease cases was calculated using a case fatality ratio, i.e., dividing the number of disease-related deaths by the disease-specific case fatality ratio (Table 1).

**Table 1:** Case fatality ratio, health services utilization (e.g., probability of seeking care conditional on being sick), and out-of-pocket direct medical costs for 20 leading causes of mortality in Ethiopia

Cause of mortality	Case fatality ratio (%)	Health services utilization (%)	Out-of-pocket direct medical costs (2013 US$)	Sources
Lower respiratory infections (LRI)	1.1 %	27 %^a^; 9 %^b^	$6^a^*; $51^b^*	[13, 25, 26]
Diarrhea	0.1	31^a^; 1^b^	5^a^*; 59^b^*	[13, 27–29]
Malaria	0.3	24^a^; 1^b^	2^a^; 4^b^	[13, 30–33]
Tuberculosis	17.0	62	49	[34, 35]
Stroke	39.7	25^a,c^; 9^b,c^	146^a^**; 343^b^**	[36–39]
Preterm birth complications	4.6	8^d^	43	Based on cesarean section provider unit cost [40] and [13, 41–44]
HIV	6.2	68	5	[45–48]
Road injury	2.3	50^c^	75	Based on surgical procedure provider unit cost [49] and [50]
Meningitis	27.0	27	66	Based on LRI inputs and [30, 51]
Ischemic heart disease	29.1	25^a,c^; 9^b,c^	338^a^**; 629^b^**	[52, 53]
Cirrhosis	33.6	2	417	Based on [55] and estimated cancer utilization, average out-of-pocket inpatient cost for breast and cervical cancer, diabetes, and cardiovascular disease
Measles	3.5	31^a^; 1^b^	5^a^; 59^b^	Based on diarrhea inputs and [54]
Whooping cough	2.8	27^a^; 9^b^	6^a^; 51^b^	Based on LRI inputs and [56]
Diabetes mellitus	21.0	25^c^	176	[57, 66]
Neonatal encephalopathy	3.9	3^e^	76	Based on intrapartum-related conditions [41–44, 67] and cesarean section provider unit cost [40]
Chronic obstructive pulmonary disease	1.1	27^a^; 9^b^	7^a^; 56^b^	Based on LRI inputs and [58]
Epilepsy	0.4	5	18	[59, 60]
Cervical cancer	66.7	2^a^; 1^b^	280^a^; 508^b^	[61–63]
Asthma	0.2	27^a^; 9^b^	7^a^; 56^b^	Based on LRI inputs and [65]
Breast cancer	54.7	2	431	[61, 63, 64]

^a^Outpatient care

^b^Inpatient care

^c^Assumptions based on expert opinion (author STM and colleagues from Ethiopia’s Federal Ministry of Health)

^d^Utilization is estimated at 8.2 %: skilled birth attendance rate of 16.4 % [41] multiplied by 0.5 according to World Health Organization expert panel estimation for effective coverage [42]

^e^Utilization is estimated at 3.3 %: skilled birth attendance rate of 16.4 % [41] multiplied by 0.2 according to World Health Organization expert panel estimation for effective coverage [42]

*Memirie ST, Metafaria ZS, Norheim OF, Levin C, Verguet S, Johansson KA. Catastrophic health expenditure and impoverishment associated with pneumonia and diarrhea treatment costs in Ethiopia. Under review

**Tolla MT, Norheim OF, Belayneh A, Amenu K, Abdisa SG, Verguet S, Johansson KA. Out-of-pocket expenditure for prevention and treatment of cardiovascular disease in general and specialized hospitals in Addis Ababa, Ethiopia. Under review

Second, we used health services utilization data from various sources, including Ethiopia’s Demographic and Health Survey [13] and additional available information from the literature. This enabled us to quantify, among the number of individuals who were ill, the fraction who used the health services specifically addressing their illness, and hence might have encountered OOP direct medical payments for that illness. For example, for diarrhea, this fraction corresponded to the percentage of patients with diarrhea seeking either outpatient or inpatient care; for TB, this fraction was derived from the case detection rate of TB (Table 1; Additional file 1: Section 1.1). For the conditions for which we did not have utilization data (e.g., ischemic heart disease, stroke, road injury), we made assumptions based on expert opinion (author STM). Services utilization included the formal health sector as expenditure data were not available for the informal sector, and this study focused on the direct payments to the formal health system.

Third, we used OOP direct medical costs data assembled through a review of the literature for each condition. Those costs included all direct medical OOP payments, including registration, diagnostic, medications, and hospital bed payments. For those conditions for which we did not have OOP direct medical costs data, we used health services provider costs data augmented by country public and private health expenditure data (national health accounts) [14], in order to derive modeled OOP direct medical costs borne by households for those conditions. Ethiopia’s latest national health accounts indicated that the fraction of healthcare expenditure borne OOP by households in 2011 was 2 % for HIV/AIDS, 14 % for malaria, 28 % for reproductive health conditions, 36 % for TB, 48 % for child health conditions, and 53 % for “other” conditions [14]. We used these percentage points to derive OOP direct medical costs from health services provider costs, when needed. For example, if the provider costs were $100 for injuries (falling into the “other” category above), then $53 would be assigned as OOP direct medical costs for injuries. All costs figures were inflated to 2013 US$ using the US consumer price index time series from the World Bank [15].

Fourth, in assigning household income, we assumed a distribution of income in the country, as there was no such distribution readily available for Ethiopia. We derived a distribution of income drawn from a simulated gamma distribution whose shape and scale parameters were based on gross domestic product per capita (2013 US$ 505, the mean of the income distribution) and Gini coefficient (0.33) [15–17]. For each occurrence of OOP direct medical costs associated with a particular condition, we assigned an annual income extracted from the income distribution. We also used the national poverty line (about 30 % of the population was estimated to be under Ethiopia’s poverty line in 2010, corresponding to an income level of about $306) [15, 18]. Subsequently, we estimated the number of households for whom the size of OOP direct medical costs would push them below the poverty line. A poverty case was counted when first household income was above the poverty line and second household income minus OOP direct medical costs was below the poverty line (Additional file 1: Section 1.2).

### Sensitivity analysis

We conducted both probabilistic and univariate sensitivity analyses. A probabilistic sensitivity analysis was conducted using Monte Carlo simulations (n = 1000 trials) where all key parameters (causes of death estimates, case fatality ratios, utilizations, OOP direct medical costs) were varied simultaneously. Uncertainty was included through sampling n values for each parameter to which either a gamma, beta, or normal distribution was assigned built on each input’s mean and standard deviation resulting in n samples. Extracting the 2.5 and 97.5 percentiles allowed the determination of 95 % uncertainty ranges (URs), which were reported with the results. Further details are given in Additional file 1: Section 2.1.

Three univariate sensitivity analyses were performed. First, we set (unrealistically) health services utilization equally to 75 % (close to the highest observed utilization among all conditions, i.e., HIV) across all 20 conditions, all other things being equal (Additional file 1: Section 2.2.1). Second, disease-specific cases, health services utilization, and the size of OOP direct medical costs were varied across the income distribution: (1) disease-specific cases were assumed to be 1.5 times higher within the poorest income quintile than in the richest income quintile; (2) utilization was set to be 2.8 times higher among the richest quintile than in the poorest quintile; (3) OOP direct medical costs were assumed to be 2.9 times higher among the richest than among the poorest (Additional file 1: Section 2.2.2). Third, we used an alternative metric to calculate medical impoverishment, i.e., we estimated the number of cases of catastrophic health costs, counting the number of households whose OOP direct medical costs would exceed 20 % of annual income (Additional file 1: Section 2.2.3). The first sensitivity analysis was meant to address the fact that those individuals who forgo healthcare because of a lack of available services are not always counted in the analysis. The second one intended to capture heterogeneity, including variations with socioeconomic status. The third sensitivity analysis was meant to illustrate the influence of the type of metric chosen for estimating medical impoverishment.

All inputs used are gathered in Table 1 and all details are collected in Additional file 1. All analyses were run using the R statistical software (www.r-project.org).

## Results

**Fig. 1:**
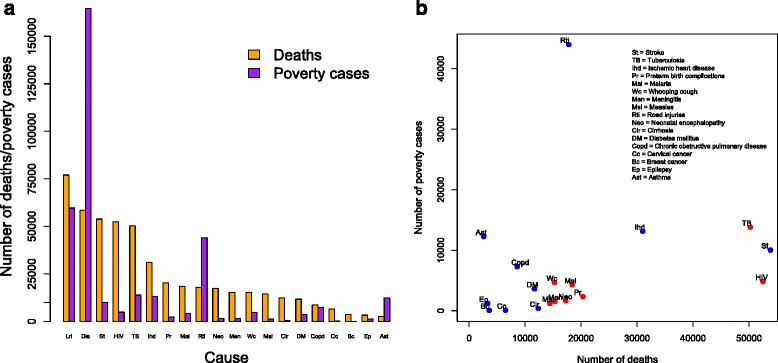
**a** Numbers of poverty cases due to out-of-pocket (OOP) direct medical costs and deaths incurred by each of 20 leading causes of mortality in Ethiopia. Lri = lower respiratory infections, Dia = diarrhea, St = stroke, TB = tuberculosis, Ihd = ischemic heart disease, Pr = preterm birth complications, Mal = malaria, Rti = road traffic injuries, Neo = neonatal encephalopathy, Men = meningitis, Wc = whooping cough, Msl = measles, Cir = cirrhosis, DM = diabetes mellitus, Copd = chronic obstructive pulmonary disease, Cc = cervical cancer, Bc = breast cancer, Ep = epilepsy, Ast = asthma. **b** Numbers of poverty cases due to OOP direct medical costs and deaths incurred by each of 20 leading causes of mortality in Ethiopia, when the two leading causes of mortality, lower respiratory infections and diarrhea, were omitted. Non-communicable diseases and injuries are indicated in blue; communicable diseases, maternal, and neonatal causes are indicated in red

The 20 causes of mortality were ranked according to the level of medical impoverishment due to OOP direct costs they incurred, allowing us to infer what inputs might have the largest impact based on cause of death, OOP costs, and utilization (Table 2; Fig. 1).

**Table 2:** Estimated total number of poverty cases, defined as condition-specific out-of-pocket (OOP) direct medical costs pushing households below the poverty line, for the base case scenario and for two sensitivity analyses ((1) 75 % utilization across all conditions and (2) key inputs including disease-specific cases, utilization, and OOP direct medical costs varying across income), and deaths incurred by each of 20 leading causes of mortality in Ethiopia. The share of poverty cases among all estimated cases and the ranking are also indicated. In brackets are presented the 95 % uncertainty ranges (URs) for the base case

Cause of mortality	Number of poverty cases due to direct OOP medical costs	Number of poverty cases due to direct OOP medical costs, 75 % utilization (1)	Number of poverty cases due to direct OOP medical costs, key inputs varying across income (2)	Number of deaths
Diarrhea	Count: 164,100 (95 % UR: 95,000–233,900) Share: 46.7 % (95 % UR: 32.4–58.1 %) Ranking: 1 (95 % UR: 1–1)	Count: 222,000 Share: 30.0 % Ranking: 1	Count: 104,000 Share: 43.2 % Ranking: 1	58,500
Lower respiratory infections	59,200 (35,800–98,400) 16.9 % (10.4–26.6 %) 2 (2–3)	165,500 22.4 % 2	38,000 15.8 % 2	77,000
Road injury	44,700 (24,200–74,800) 12.8 % (6.8–20.5 %) 3 (2–3)	66,800 9.0 % 3	32,000 13.3 % 3	17,800
Tuberculosis	13,600 (7400–23,100) 3.9 % (2.1–6.8 %) 4 (4–8)	16,300 2.2 % 11	8700 3.6 % 6	31,000
Ischemic heart disease	13,100 (8400–20,300) 3.7 % (2.2–6.2 %) 5 (4–7)	39,500 5.3 % 4	16,300 30.0 % 4	50,200
Asthma	12,400 (7200–20,600) 3.5 % (2.0–6.1 %) 6 (4–8)	33,700 4.6 % 6	7700 3.2 % 7	2600
Stroke	9900 (6300–16,000) 2.8 % (1.7–4.7 %) 7 (4–9)	29,900 4.0 % 7	11,300 4.7 % 5	53,800
Chronic obstructive pulmonary disease	7300 (4600–12,200) 2.1 % (1.2–3.7 %) 8 (5–10)	20,200 2.7 % 9	4700 2.0 % 8	8600
HIV	4,900 (2,500–8400) 1.4 % (0.7–2.5 %) 9 (8–13)	5400 0.7 % 16	3000 1.2 % 7	52,500
Whooping cough	4800 (2800–7700) 1.4 % (0.7–2.3 %) 10 (8–13)	13,000 1.8 % 14	3000 1.2 % 10	15,300
Malaria	4200 (2400–7200) 1.2 % (0.6–2.2 %) 11 (8–13)	13,000 1.8 % 13	2,600 1.1 % 12	18,400
Diabetes mellitus	3700 (2000–5900) 1.0 % (0.5–1.8 %) 12 (9–14)	10,600 1.4 % 15	3500 1.4 % 9	11,700
Preterm birth complications	2,400 (1,200–4,200) 0.7 % (0.3–1.3 %) 13 (10–16)	22,100 3.0 % 8	1500 0.6 % 13	17,300
Neonatal encephalopathy	1600 (1000–3000) 0.5 % (0.3–0.9 %) 14 (12–17)	38,700 2.7 % 5	1200 0.5 % 14	12,300
Meningitis	1500 (800–2700) 0.4 % (0.2–0.8 %) 15 (13–17)	4200 0.6 % 17	1000 0.4 % 15	15,300
Measles	1200 (700–2000) 0.3 % (0.2–0.6 %) 16 (14–17)	2800 0.4 % 19	700 0.3 % 16	14,400
Epilepsy	1100 (600–2100) 0.3 % (0.2–0.6 %) 17 (14–17)	17,300 2.3 % 10	700 0.3 % 17	3300
Cirrhosis	400 (200–600) 0.1 % (0.1–0.2 %) 18 (18–18)	13,700 1.8 % 12	500 0.2 % 18	12,300
Cervical cancer	<100 (0–100) 0.0 % (0.0–0.0 %) 19 (19–20)	3200 0.4 % 18	< 100 0.0 % 19	6500
Breast cancer	< 100 (0–100) 0.0 % (0.0–0.0 %) 20 (19–20)	2500 0.3 % 20	< 100 0.0 % 20	3600

The three top drivers of impoverishment, defined as OOP direct medical costs pushing households below the poverty line, were diarrhea, with approximately 164,000 poverty cases (95 % UR: 95,000–233,900; approximately 47 % (95 % UR: 32–58 %) of all 350,000 cases estimated); lower respiratory infections, with approximately 59,000 cases (35,800–98,400; 17 % (10–27 %) of all cases); and road injuries, with approximately 45,000 cases (24,200–74,800; 13 % (7–20 %) of all cases). The three bottom conditions were breast cancer, with approximately 50 poverty cases (30–100, < 0.1 % of all cases); cervical cancer, with approximately 70 cases (40–120, < 0.1 % of all cases); and cirrhosis with approximately 390 cases (200–600, 0.1 % of all cases) (Table 2). All three bottom conditions had very low utilization rates (2 %).

We observed substantial impoverishment due to OOP direct medical costs for diarrhea and lower respiratory infections (Fig. 1a). The distribution of medical impoverishment by cause became much tighter (road injuries, TB, and ischemic heart disease, then representing 35 %, 11 %, and 10 % of all remaining poverty cases, respectively), when both diarrhea and lower respiratory infections were removed from the analysis. Moreover, we observed that a large number of non-communicable diseases (e.g., ischemic heart disease, asthma, stroke) led to substantial medical impoverishment (Fig. 1b). Some of the important drivers of the medical impoverishment findings seemed to be health services utilization and the number of illness cases, much less so though when, again, diarrhea and lower respiratory infections were removed. On the other hand, OOP direct medical costs seemed less of a driver.

**Fig. 2:**
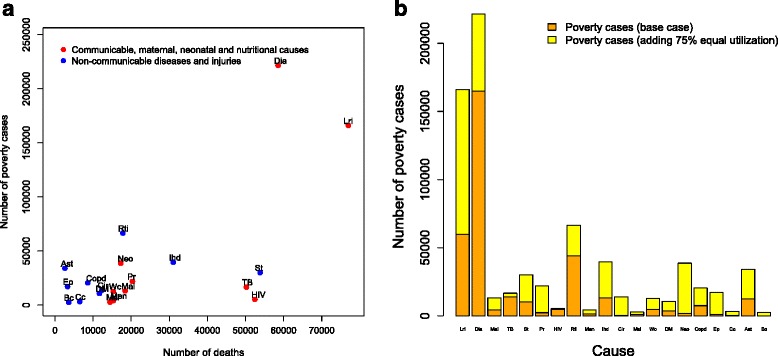
**a** Number of poverty cases due to direct out-of-pocket (OOP) medical costs and number of deaths incurred by each of 20 leading causes of mortality in Ethiopia, when health services utilization was hypothetically set at 75 % across all conditions. Non-communicable diseases and injuries are indicated in blue; communicable diseases, maternal, and neonatal causes are indicated in red. **b** Number of poverty cases due to direct OOP medical costs by each of 20 leading causes of mortality in Ethiopia, in the base case (orange) and when adding health services utilization set at 75 % across all conditions (yellow). Lri = lower respiratory infections, Dia = diarrhea, St = stroke, TB = tuberculosis, Ihd = ischemic heart disease, Pr = preterm birth complications, Mal = malaria, Rti = road traffic injuries, Neo = neonatal encephalopathy, Men = meningitis, Wc = whooping cough, Msl = measles, Cir = cirrhosis, DM = diabetes mellitus, Copd = chronic obstructive pulmonary disease, Cc = cervical cancer, Bc = breast cancer, Ep = epilepsy, Ast = asthma

When we unrealistically set health services utilization to 75 % across all conditions, in other words, when access barriers were fully removed, expectedly, the magnitude of impoverishment tied to OOP direct medical costs was substantially augmented (Table 2; Fig. 2). This confirmed that low utilization would impact on the medical impoverishment findings. We observed a reordering of rankings beyond the first three leading causes of medical impoverishment (Table 2; Fig. 2a), and could point to the contribution of reduced access to care in the estimated small numbers of poverty cases for several conditions in the base case (Fig. 2b).

**Fig. 3:**
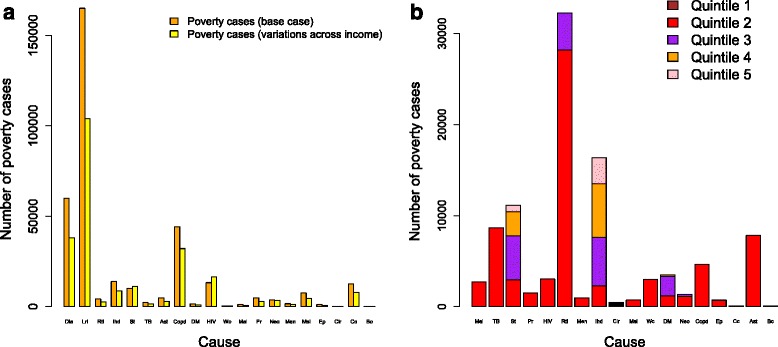
**a** Number of poverty cases due to direct out-of-pocket (OOP) medical costs by each of 20 leading causes of mortality in Ethiopia, in the base case (orange) and when key inputs (e.g., disease-specific cases, utilization, OOP direct medical costs) were varied across the income distribution (yellow). **b** Number of poverty cases due to direct OOP medical costs by each of 20 leading causes of mortality in Ethiopia, when key inputs (e.g., disease-specific cases, utilization, OOP direct medical costs) were varied across the income distribution, including the repartition among income quintiles. Lri = lower respiratory infections, Dia = diarrhea, St = stroke, TB = tuberculosis, Ihd = ischemic heart disease, Pr = preterm birth complications, Mal = malaria, Rti = road traffic injuries, Neo = neonatal encephalopathy, Men = meningitis, Wc = whooping cough, Msl = measles, Cir = cirrhosis, DM = diabetes mellitus, Copd = chronic obstructive pulmonary disease, Cc = cervical cancer, Bc = breast cancer, Ep = epilepsy, Ast = asthma

When we varied key inputs (e.g., disease cases, utilization, OOP direct medical costs) across the income distribution, we found an overall reduction in the estimated burden of medical impoverishment and almost all conditions had such a decrease (Fig. 3a); yet, there were only slight changes in rankings (Table 2). Further, we examined the distribution of poverty cases incurred per condition per income quintile (Fig. 3b). We found that no poverty cases would occur among the bottom quintile due to the use of the poverty case metric (i.e., those already under the poverty line were not counted). We observed a distinct distribution of medical impoverishment across income quintiles depending on the condition: the conditions with large OOP direct medical costs (e.g., stroke, ischemic heart disease) had all four quintiles impacted, contrary to the conditions with small OOP direct medical costs (e.g., asthma, COPD).

Finally, when we used an alternative metric of cases of catastrophic health costs (Additional file 1: Section 2.2.3), there was a change in the medical impoverishment rankings. Notably, all income quintiles would now be impacted by medical impoverishment (including those previously under the poverty line).

## Discussion

The purpose of this paper was to present a preliminary attempt to systematically estimate medical impoverishment by cause, and the difficulties inherent in making such assessments.

In our case study of Ethiopia, we found that medical impoverishment was tied to illness cases and health services utilization. Cancers presented high OOP costs, but the corresponding population-level medical impoverishment was, in fact, small because countrywide cases of cancer are low, and utilization difficult (Ethiopia has only one cancer treatment center for the whole country, located in a specialized hospital in the capital city of Addis Ababa). However, when we omitted the two leading causes of mortality (lower respiratory infections and diarrhea), we observed little overlap between disease-specific mortality and impoverishment due to OOP direct medical costs.

This approach to medical impoverishment estimation could, with more local data, enable policymakers to take health-related payment criteria into account when designing the architecture of health systems. Financial protection is central to achieving equity and fairness, and a key component of UHC. While economic evaluations have tended to focus on estimating health improvements per dollar spent [19], newer methods, such as extended cost-effectiveness analysis (ECEA) [11, 20], allow the quantification of financial protection that can be purchased by health policies. A systematic assessment like ours aims at identifying both the proportion of individuals impoverished by OOP direct medical costs and the causes of such impoverishment. This can provide an evidence base for efficient policy and planning. For instance, in 2011 in Ethiopia, the main reason households faced medical impoverishment was expenditure on drugs [21], which suggested the need for appropriate prepayment mechanisms for accessing healthcare. The government began adopting community-based health insurance and developing social health insurance [22]. A systematic approach quantifying the factors leading to medical impoverishment can point to the causes which social programs should address in order to improve financial protection and equity for all.

Nonetheless, our preliminary attempt has several important limitations. First, we only describe the unidirectional impact of illness on OOP medical impoverishment, and do not include the impact of poverty on health. Second, we deliberately focused on OOP direct medical costs. Yet, there are a number of other factors in impoverishment. Transport costs and indirect costs, including loss of earnings, may contribute significantly to poverty. Illness-related poverty may be correlated with chronic conditions (e.g., epilepsy) among productive adults, leading to more wages lost than acute conditions (e.g., diarrhea). The illness and death of wage earners may impoverish a family over a long time; as this suggests, which individual is impacted in the family (child vs. bread-winner) would matter. However, we were prevented from including these considerations due to the lack of reliable data across conditions. Third, our analysis critically points to the importance of health services utilization data and the inadequacy of current data at the national and global levels. Further utilization data for a large number of conditions is urgently needed. Before new data becomes available, an interim solution would be to estimate utilization for selected groups of conditions (e.g., childhood conditions, major infectious diseases, cancers), depending on incidence, service availability, and recorded patient loads for services in countries. The scarcity of data, including the limited data on utilization but also on OOP costs or private providers (e.g., traditional healers), influenced the choice of our parsimonious modeling approach, relying on secondary inputs and focusing on a limited number of conditions. We restricted our study to leading causes of mortality (and could not include causes of morbidity) as a systematic assessment by cause was solely available for mortality [12]. Yet, by examining high mortality conditions only, our results may be biased against chronic conditions for which payments may be repeated across long time periods. Similarly, for some conditions, we relied on macro-level indicators (e.g., sources of funding for health) and on provider costs to derive OOP costs even though OOP costs are not necessarily based on providers’ costs. We did not include heterogeneity (e.g., geographical variation) in the model due to lack of data. For all these reasons, we preferred a simple framework. We acknowledge that many inputs (e.g., mortality, utilization, OOP costs) present substantial uncertainty; our findings should be interpreted with caution and future work should carefully contextualize our analysis. Fourth, we chose to represent disease-specific impoverishment due to OOP direct medical costs in estimating a headcount of poverty cases in spite of the shortcomings of such threshold-based metrics [2]. Alternative metrics include the estimation of cases of catastrophic health costs [4], insurance risk premiums [20], or measures of downside risk arising from uninsured expenditure [23]. We chose the cases of poverty metric for its simplicity, and pursued a sensitivity analysis estimating cases of catastrophic health costs. Further, OOP payments were simply compared to income, not accounting for household borrowing capacity, which could lead to underestimation of coping mechanisms and overestimation of impoverishment [24].

Finally, our approach may mask the importance of the size of OOP costs and the lack of access to care. Some conditions may lead to large numbers of poverty cases because care is available and hence incurs OOP costs (e.g., road injuries), whereas others may lead to few poverty cases as care is rarely available and thus little in OOP costs can be expected (e.g., cancers). A focus on current medical impoverishment and utilization patterns is not able to distinguish between these two situations. One of our sensitivity analyses is set at 75 % utilization for all conditions in order to acknowledge this limitation. Studying medical impoverishment must be undertaken jointly with the contextual examination of available health services. As greater medical impoverishment may be driven by greater service availability, it would be inadvisable to prioritize services on the basis of greater medical impoverishment only. We emphasize herein the potential financial catastrophes of private financing for health services.

## Conclusion

Our intentionally simple approach presents a preliminary attempt to systematically consider the catastrophic consequences of OOP direct medical payments in low- and middle-income countries and the difficulties in such an undertaking. We hope it can be a departure point for substantial empirical and analytical improvements. Our framework represents a stepping stone toward highlighting the importance of the financial protection benefits that could be procured by the scale-up of health interventions. Medical impoverishment findings complemented by the effectiveness of benefits packages and social safety nets can help estimate how much medical impoverishment could be averted by the scale-up of health policies.

## Additional file

Additional file 1: 1. Inputs and methods for the estimation of the burden of impoverishment due to out-of-pocket direct medical costs. 2. Sensitivity analyses. (PDF 759 kb)
