# Supplementary material for: Assessing the burden of medical impoverishment by cause: a systematic breakdown by disease in Ethiopia
Source: BMC Med. 2016 Oct 21;14:164. doi: 10.1186/s12916-016-0697-0 (PMC5075208; doi:10.1186/s12916-016-0697-0)
Supplement: Additional file 1: — 1. Inputs and methods for the estimation of the burden of impoverishment due to out-of-pocket direct medical costs. 2. Sensitivity analyses. (PDF 759 kb) [file 12916_2016_697_MOESM1_ESM.pdf]

Additional file 1

Assessing the burden of medical impoverishment by cause: a systematic breakdown by disease in Ethiopia

by Stéphane Verguet, Solomon Tessema Memirie, Ole Frithjof Norheim

1. Inputs and methods for the estimation of the burden of impoverishment due to out-of-pocket direct medical costs.

1.1. Derivation of key inputs

Table S1. Detailed derivation of key epidemiological and cost inputs used in the estimation of Ethiopia's burden of impoverishment due to out-of-pocket direct medical costs.

Cause of mortality	Explanation
Lower respiratory infections (LRI)	• 27% of sick patients seek care [1] including 18.3% seeking outpatient care and 8.7% seeking inpatient care [2].<br>• Case fatality ratio of 1.1% derived from the ratio of the numbers of annual LRI cases (3,367,561) and annual LRI deaths (37,269) for 0-4 year-olds for Ethiopia in 2010 [3].<br>• Out-of-pocket cost estimates extracted from [4].
Diarrhea	• 31% of sick patients seek care [1] including 30.5% seeking outpatient care and 0.5% [5] seeking inpatient care.<br>• Case fatality ratio of 0.09% derived from the ratio of the numbers of annual diarrhea cases (3.04*13,914,000=42,298,560) [6,7] and annual diarrhea deaths (38,535) [3] for 0-4 year-olds for Ethiopia in 2010.<br>• Out-of-pocket cost estimates extracted from [4].
Malaria	• 24% of sick patients seek care [1] including 23.3% seeking outpatient care and 0.7% seeking inpatient care, as derived from the ratio of outpatient malaria cases to inpatient malaria cases (23.4 per 1,000 and 6.4 per 10,000, respectively from [8]).<br>• Case fatality ratio of 0.3% derived from the ratio of the numbers of annual malaria cases (163,000,000) and annual malaria deaths (528,000) for Africa in 2013 [9].<br>• Out-of-pocket outpatient cost estimate of US$2003 1.40 extracted from [10] inflated to $1.80 (US$ 2013) using U.S. consumer price index (CPI) [11].<br>• Out-of-pocket inpatient cost estimate derived from: provider cost of US$2009 29 [12] inflated to $32 (US$ 2013) using U.S. CPI [11], then multiplied by 0.14 (share of malaria costs borne out-of-pocket by households [13]) leading to $4.4 (US$ 2013).

Tuberculosis (TB)	• 62% of sick patients seek care, derived from the case detection rate of all forms of TB for Ethiopia [14].<br>• Case fatality ratio of 17.0% derived from the ratio of TB mortality (30,000+5,600 deaths) and TB incidence (210,000 cases) in 2010 for Ethiopia [14].<br>• Out-of-pocket cost derived from: provider cost of US$2006 116.5 [15] inflated to $135 (US$2013) using U.S. CPI [11], then multiplied by 0.36 (share of TB costs borne out-of-pocket by households [13]) leading to $49 (US$ 2013).
Stroke	• 25% of sick patients seek care: 16.3% seeking outpatient care and 8.7% seeking inpatient care (based on expert opinion).<br>• Case fatality ratio of 39.7% derived from the average of three case fatality ratios: 28% [16], 114 divided by 244 = 46.7% [17], and 44.5% [18].<br>• Out-of-pocket cost estimates extracted from [19].
Preterm birth complications	• Utilization of 8.2% derived from skilled birth attendance rate of 16.4% [20] multiplied by 0.5 according to the World Health Organization expert panel estimation for effective coverage [21].<br>• Case fatality ratio of 4.6% derived from: incidence of preterm birth of 14.3% (Eastern Africa) [22] multiplied by 15,537,000 / 5 births [6] = 444,358 preterm births and 20,335 deaths from preterm birth complications in Ethiopia in 2013 [23].<br>• Out-of-pocket cost derived from: provider cost for Cesarean section unit cost of US$2005 132.7 [24] inflated to $158 (US$ 2013) using U.S. CPI [11], then multiplied by 0.27 (share of reproductive health costs borne out of pocket by households [13]) leading to $43 (US$ 2013).
HIV	• Utilization of 68% corresponding to antiretroviral therapy coverage [25].<br>• Case fatality ratio of 6.2% derived from 760,000 people living with HIV in 2012 and 47,000 HIV-related deaths in 2013 in Ethiopia [25].<br>• Out-of-pocket cost derived from: (i) [26] with provider cost of US$2004 265 of which 70% was for antiretroviral (ARV) drugs so non-ARV drug component of US$2004 79.5 inflated to US$2013 98 using U.S. CPI; (ii) [27] with provider cost of US$2010 186 of which $103 was for ARV drugs leading to $110 for ARV and $89 for non-ARV (US$ 2013) using U.S. CPI; (iii) [28] with US$2003 75 and 67% of which was for ARV drugs leading to US$2013 295 for non-ARV drugs. We used $110 for ARV drugs and the average of $295, $98 and $89 for non-ARV drugs leading to a provider cost of $271 (US$ 2013). Finally, we multiplied by 0.02 the share of HIV costs borne out of pocket by households [13] and eventually obtained US$2013 5 for out-of-pocket cost.
Road injury	• Utilization of 50% (based on expert opinion).<br>• Case fatality ratio of 2.3% derived from the ratio of estimated number of injuries (355,719+3,267,240) and estimated number of injury-related deaths (83,614) for Eastern sub-Saharan Africa [29].<br>• Out-of-pocket cost derived from: provider cost for surgical procedure of ETB2008 1,125 [30] with 2008 exchange rate (1$ = 9.2ETB) and inflated to $141 (US$ 2013) using U.S. CPI [11], then multiplied by 0.53 (share of "other" costs borne out of pocket by households [13]) leading to $75 (2013 US$).
Meningitis	• Utilization of 27% based on LRI treatment seeking [1].<br>• Case fatality ratio of 27.0% extracted from [31].

	• Out-of-pocket estimate derived from: provider cost of US$2009 126 [32] inflated to $137 (2013 US$) using U.S. CPI [11], then multiplied by 0.48 (share of child health costs borne out of pocket by households [13]) leading to $66 (2013 US$).
Ischemic heart disease	• 25% of sick patients seek care: 16.3% seeking outpatient care and 8.7% seeking inpatient care (based on expert opinion).<br>• Case fatality ratio of 29.1% derived from the average of two case fatality ratios: 28.4% = (471+87)/1965 [33] and 29.7% = 57/191.8 [34].<br>• Out-of-pocket cost estimates extracted from [19].
Cirrhosis	• Overall utilization of 1.7% taken the same as for breast and cervical cancer (based on expert opinion).<br>• Case fatality ratio extracted from [35].<br>• Out-of-pocket cost derived as the average of out-of-pocket inpatient cost for ischemic heart disease, stroke, cervical cancer, breast cancer, and diabetes.
Measles	• Utilization and out-of-pocket costs similar to the diarrhea inputs (based on expert opinion).<br>• Case fatality ratio of 3.5% extracted from [36] corresponding to the average of 3% and 4%.
Whooping cough	• Utilization and out-of-pocket costs similar to the LRI inputs (based on expert opinion).<br>• Case fatality ratio of 2.8% extracted from [37].
Diabetes mellitus	• Utilization (based on expert opinion).<br>• Case fatality ratio of 21.0% extracted from [38].<br>• Out-of-pocket cost derived from: provider cost for treatment per year of Int$2005 1132.4 [39] leading to $281 (2005US$) using 2005 exchange rate ($1 = $PPP4.0), and using U.S. CPI [11], leading to US$2013 335, then multiplied by 0.53 (share of the category "other" costs borne out of pocket by households [13]) eventually leading to $176 (2013 US$).
Neonatal encephalopathy	• Utilization of 3.3% derived from skilled birth attendance rate of 16.4% [20] multiplied by 0.2 according to the World Health Organization expert panel estimation for effective coverage [21].<br>• Case fatality ratio of 3.9% derived from: incidence of preterm birth of 14.3% (Eastern Africa) [22] multiplied by 15,537,000 / 5 births [6] = 444,358 preterm births and 17,270 deaths from neonatal encephalopathy in Ethiopia in 2013 [23].<br>• Out-of-pocket cost derived from: provider cost for Cesarean section unit cost US$2005 132.7 [24] inflated to $158 (2013 US$) using U.S. CPI [11], then multiplied by 0.48 (share of child health costs borne out of pocket by households [13]) leading to $76 (2013 US$).
Chronic obstructive pulmonary disease (COPD)	• Utilization similar to the LRI inputs (based on expert opinion).<br>• Case fatality ratio of 1.1% derived from: 1% and 3.5% prevalence among 20 years and older in Ethiopia [40] leading to 0.01*42,627,392 cases [6] and 0.035*42,627,392 [6]; 0.6% among all age groups in Ethiopia [41] leading to 0.006*89,859,000 cases, which leads to an average of 749,135 COPD cases compared to 8,598 deaths for 2013 in Ethiopia [23].<br>• Out-of-pocket estimate derived from [11] as inpatient 2013US$ 51 and outpatient 2013US$ 6 then adjusted by 0.53/0.48 (the ratio of the share of the category "other" costs borne out of pocket by households and the share of child health costs borne out of pocket by households [13]) leading to $56 (inpatient,

	2013 US$) and $7 (outpatient, 2013 US$).
Epilepsy	• Utilization of 5% extracted from [42].<br>• Case fatality ratio of 0.37% derived from prevalence of 9.39 per 1,000 for Ethiopia [43] and multiplied by population of 93,476,282 leading to 877,742 cases compared with 3,280 deaths [23].<br>• Out-of-pocket estimate derived from: provider cost for antiepileptic treatment from 0.01*94,100,756 = 941,008 people with epilepsy in Ethiopia, 75% coverage and a cost of 22.16 million dollars (assumed 2010 USD) [42] leads to US$2010 31 per person inflated [11] to 2013 US$ 34; multiplied by 0.53 the share of "other" borne out of pocket by household leads to $18 (US$ 2013).
Cervical cancer	• Overall utilization of 1.7% estimated from: the number of annual cancer patients (2,000 adults and 200 children) at Tikur Anbessa hospital [44] and estimated annual prevalence number of cancer cases in Ethiopia (129,962) [41]; 1.1% inpatient and 0.6% outpatient as 53.7% of patients in [45] were inpatients.<br>• Case fatality ratio of 66.7% derived from 7,095 cases and 4,732 deaths [41].<br>• Out-of-pocket cost: outpatient derived from ETB1002.9 + ETB3148.4 (2011) at 2011 exchange rate ($1 = ETB16.14) [11] inflated by U.S. CPI [11] leading to US$2013 280; inpatient derived from ETB1002.9 + ETB3148.4 (2011) + ETB3544.1 (2011) inflated to US$2013 508.
Asthma	• Utilization similar to the LRI inputs (based on expert opinion).<br>• Case fatality ratio of 0.18% derived from prevalence of 2.00% in Ethiopia [46] multiplied by population of 93,476,282 leading to 1,869,526 cases compared with 2,616 deaths [23].<br>• Out-of-pocket estimate derived from [4] as inpatient 2013US$ 51 and outpatient 2013US$ 6 then adjusted by 0.53/0.48 (the ratio of the share of the category "other" costs borne out of pocket and the share of child health costs borne out of pocket [13]) leading to $56 (inpatient, 2013 US$) and $7 (outpatient, 2013 US$).
Breast cancer	• Overall utilization of 1.7% estimated from: the number of annual cancer patients (2,000 adults and 200 children) at Tikur Anbessa hospital [44] and estimated annual prevalence number of cancer cases in Ethiopia (129,962) [41].<br>• Case fatality ratio of 54.7% as derived from 12,956 cases and 7,089 deaths [41].<br>• Out-of-pocket estimate derived from: provider cost of 602$ (2000) [47] inflated to USD2013 814 using the U.S. CPI [11], then adjusted by 0.53 the share of the category "other" costs borne out of pocket by households [13] leading to $431 (2013 US$).

1.2. Mathematical methods used in the analysis

This subsection describes the mathematical methods used for assessing impoverishment due to out-of-pocket (OOP) direct medical costs per condition.

Denote the household income y, the total number of households H, the condition J, and the corresponding total number of cases Ca_J. Let p_J denote the probability of seeking care for the treatment of the condition J conditional on having J, and c_J the OOP direct medical costs for the treatment of J.

Before condition J occurred, we assumed the income was y. When there is exposure to the risk of condition J, the expected value of income becomes:

$$E_J(y) = \frac{Ca_J}{H}\left[p_J(y - c_J\ ) + \left(1 - p_J\ \right)y\right] + (1 - \frac{Ca_J}{H})y\ . \qquad (1)$$

We then estimated the number of poverty cases attributed to OOP direct medical costs due to condition J. To do so, we first counted the number of households for which OOP direct medical costs c_J would be incurred, which corresponded to $Ca_J * p_J$ households. Second, among those $Ca_J * p_J$ households (out of the total H), we counted those households for which: (i) $y > P_l$, and (ii) $y - c_J < P_l$, where P_l was the country poverty line. We used the national poverty line P_l (around \$306 or about 30% of the population was estimated to be under Ethiopia's poverty line in 2010) [11,48].

Concerning y, as there was no income distribution readily available for Ethiopia, we derived a distribution of income drawn from a simulated gamma distribution whose shape and scale parameters were based on gross domestic product per capita (2013US\$ 505, the mean of the distribution) and Gini coefficient (0.33) (both available from the World

Bank's World Development Indicator database) [11,49,50]. For each occurrence of OOP direct medical costs associated with a particular condition J among $Ca_J * p_J$ households, we sampled an annual income y extracted from the income distribution. Subsequently, we could estimate the number of households (among those $Ca_J * p_J$ households) for whom the size of OOP direct medical costs c_J would push them under the poverty line P_l. A poverty case was counted when first household income was above the poverty line ($y > P_l$) and second household income minus OOP direct medical costs was below the poverty line ($y - c_J < P_l$).

2. Sensitivity analyses

2.1. Probabilistic sensitivity analysis

We conducted a Monte Carlo probabilistic sensitivity analysis to estimate aggregate uncertainty from key inputs. Parameters were given values using probability distributions (details are given in Table S2).

Table S2. Probability distributions of the key inputs for the analysis.

Input	Parameters
Number of deaths, per condition	Normal with: Mean (table 2, main text) and SD as given by the Global Burden of Disease study [23]
Out-of-pocket direct medical cost, per condition ($)	Gamma with: Mean (table 1, main text) and SD equal to 1/3 of Mean
Utilization, per condition (%)	Beta with: Mean (table 1, main text) and SD equal to 1/3 of Mean
Case fatality ratio, per condition (%)	Beta with: Mean (table 1, main text) and SD equal to 1/3 of Mean

SD, standard deviation.

2.2. Univariate sensitivity analyses

We pursued three univariate sensivity analyses, which we describe in detail below.

2.2.1. Impact of health services utilization

We set health services utilization to 75% equally across all twenty conditions (e.g. tuberculosis shifted from 62% to 75% utilization). 75% was closest to 68%, the HIV coverage, which was the highest utilization rate in the base case (table 1 in the main text). For the conditions that implied inpatient and outpatient care (e.g. diarrhea), we set outpatient care utilization to 75% (e.g. from 30.5% to 75% for diarrhea), and then scaled inpatient care utilization proportionally to the base case ratio between inpatient and

outpatient care utilization (e.g. 0.5% divided by 30.5% for diarrhea, led to 75*0.5/30.5 = 1.3% for the scaled utilization of diarrhea inpatient care).

2.2.2. Impact of key inputs varying across income

We varied three key inputs across the income distribution (i.e. by income quintile): (i) disease-specific cases; (ii) health services utilizations; and (iii) OOP direct medical costs.

For disease-specific cases, due to lack of data per condition, we tentatively used the mean of the ratios between the bottom and top wealth quintiles of: under-five mortality (137 and 86 per 1,000 live births, respectively), prevalence of acute respiratory infections (7.6% and 4.0%, respectively), prevalence of fever (18.5% and 15.5%, respectively), and prevalence of diarrhea (15.0% and 11.2%, respectively). These four indicators were extracted from the Demographic and Health Survey [1] and were among the sole disease-specific indicators we could find available per wealth quintile for Ethiopia. We obtained a mean ratio of 1.51 between poorest and richest, which we distributed evenly across all income quintiles (1.51, 1.38, 1.25, 1.13, 1.00; from the poorest to the richest income quintile).

For health services utilization, due to lack of data per condition, we used the mean ratios between the bottom and top wealth quintiles of: coverage of 3rd dose of Diphtheria-Tetanus-Pertussis (26.0% and 61.5%, respectively), utilization for treatment of diarrhea (22.4% and 53.0%, respectively), utilization for treatment of fever (16.0% and 40.4%, respectively), and utilization for treatment of acute respiratory infections (15.5% and 61.7%, respectively). These four indicators were extracted from the Demographic and Health Survey [1] and were among the sole utilization-specific indicators we could find

available per wealth quintile for Ethiopia. We obtained a mean ratio of 2.81 between richest and poorest, which we distributed evenly across all income quintiles (1.00, 1.45, 1.90, 2.36, 2.81; from the poorest to the richest income quintile).

For the OOP direct medical costs, no information was available across socioeconomic groups. Hence, we tentatively used as a proxy the ratios between private and public care of OOP direct medical costs for the treatments of mild diarrhea, severe diarrhea, mild pneumonia, and severe pneumonia, as empirically estimated by Memirie and colleagues [4]. This led to a mean ratio of 2.94, which we distributed evenly across all income quintiles (1.00, 1.48, 1.97, 2.45, 2.94; from the poorest to the richest income quintile).

As described above (section 1.2), we assigned an annual household income drawn from a simulated gamma distribution of income whose shape and scale parameters were based on the country's gross domestic product per capita and Gini coefficient [11,48,50]. This allowed us to define an annual income for each household impacted by condition J. The household income was also used to define the income quintile q into which each household belonged.

Subsequently, based on the key inputs described above, per income quintile q, and per condition J we distributed: disease-specific cases $Ca_{J,q}$, health services utilizations $p_{J,q}$, and OOP direct medical costs $c_{J,q}$. We also sampled $p_{J,q} * Ca_{J,q}$ incomes y_q from the income quintile q of the simulated gamma distribution of income, and then could estimate for each condition J and each income quintile q, the number of household poverty cases counting the number of households for which first $y_q > P_l$ and second $y_q - c_{J,q} < P_l$.

2.2.3. Impact of the use of an alternative metric for the estimation of impoverishment due to out-of-pocket direct medical costs

We used an alternative metric for quantifying medical impoverishment and estimated the number of incurred cases of catastrophic health costs, per condition. It corresponded to counting the number of households for which, per condition J, the OOP direct medical costs c_J would exceed a given threshold Th (e.g. 20%) of income y. In other words, sampling from the simulated gamma distribution of income, and using the same approach as described above, we counted the number of households (among those $Ca_J * p_J$ households) for which: $c_J > Th * y$.

The results of this sensitivity analysis are displayed in Table S3 and Figure S1.

Table S3. Total number of cases of catastrophic health costs (with a threshold of 20% of income) due to out-of-pocket (OOP) direct medical costs incurred by each of twenty leading causes of mortality in Ethiopia, for the base case scenario (table 2, main text). The share of cases of catastrophic health costs among all estimated cases and the ranking are also indicated.

Cause of mortality	Number of catastrophic cases due to direct OOP medical costs	Number of poverty cases due to direct OOP medical costs (table 2, main text)
Diarrhea	Count: 100,800<br>Share: 17·6%<br>Ranking: 3	Count: 164,100<br>Share: 46·7%<br>Ranking: 1
Lower respiratory infections	135,200<br>23·5%<br>2	59,200<br>16·9%<br>2
Road injury	154,400<br>26·9%<br>1	44,700<br>12·8%<br>3
Tuberculosis	36,700<br>6·4%<br>4	13,600<br>3·9%<br>4
Ischemic heart disease	26,600<br>4·6%<br>7	13,100<br>3·7%<br>5
Asthma	30,000<br>5·3%<br>5	12,400<br>3·5%<br>6
Stroke	29,400<br>5·1%<br>6	9,900<br>2·8%<br>7
COPD	17,900<br>3·1%<br>8	7,300<br>2·1%<br>8
HIV	800<br>0·1%<br>15	4,900<br>1·4%<br>9
Whooping cough	10,500<br>1·8%<br>10	4,800<br>1·4%<br>10

Malaria	200<br>< 0·1%<br>18	4,200<br>1·2%<br>11
Diabetes mellitus	12,300<br>1·8%<br>9	3,700<br>1·0%<br>12
Preterm birth complications	5,800<br>1·0%<br>12	2,400<br>0·7%<br>13
Neonatal encephalopathy	6,000<br>1·0%<br>11	1,600<br>0·5%<br>14
Meningitis	5,000<br>0·9%<br>13	1,500<br>0·4%<br>15
Measles	700<br>0·1%<br>17	1,200<br>0·3%<br>16
Epilepsy	1,100<br>0·2%<br>14	1,100<br>0·3%<br>17
Cirrhosis	700<br>0·1%<br>16	400<br>0·1%<br>18
Cervical cancer	200<br>< 0·1%<br>19	< 100<br>0·0%<br>19
Breast cancer	100<br>< 0·1%<br>20	< 100<br>0·0%<br>20

COPD, chronic obstructive pulmonary disease.

Figure S1.

(a) Numbers of cases of catastrophic health costs (CHC) (with a threshold of 20% of income) due to out-of-pocket direct medical costs and deaths incurred by each of twenty leading causes of mortality in Ethiopia.

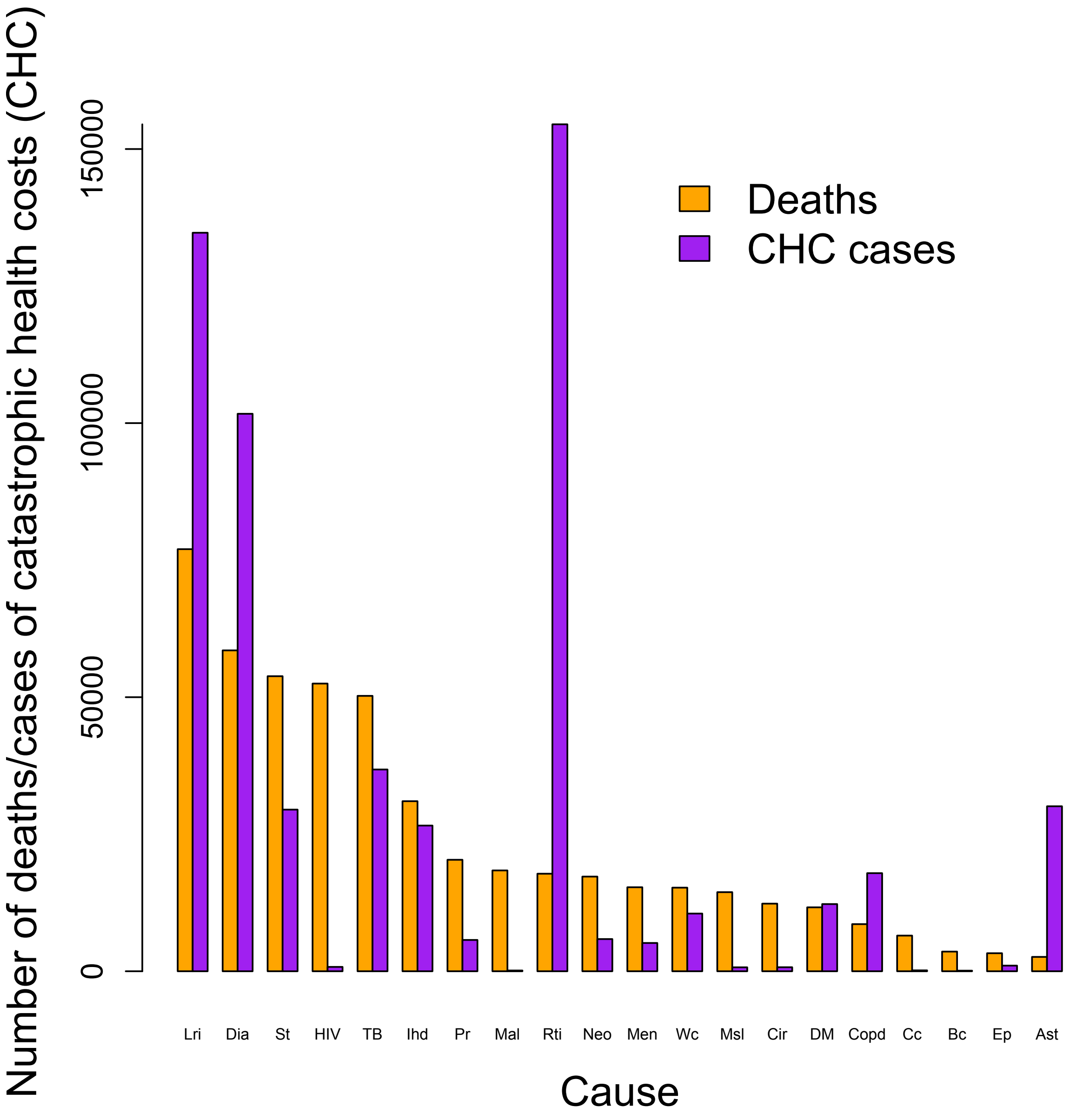

Note: Lri = lower respiratory infections, Dia = diarrhea, St = stroke, TB = tuberculosis, Ihd = ischemic heart disease, Pr = preterm birth complications, Mal = malaria, Rti = road traffic injuries, Neo = neonatal encephalopathy, Men = meningitis, Wc = whooping cough, Msl = measles, Cir = cirrhosis, DM = diabetes mellitus, Copd = chronic obstructive pulmonary disease, Cc = cervical cancer, Bc = breast cancer, Ep = epilepsy, Ast = asthma.

(b) Numbers of cases of catastrophic health costs (with a threshold of 20% of income) due to out-of-pocket direct medical costs and deaths incurred by each of twenty leading causes of mortality in Ethiopia, when the two leading causes of mortality, lower respiratory infections and diarrhea, were omitted. Non-communicable diseases and injuries are indicated in blue; communicable diseases, maternal and neonatal causes are indicated in red.

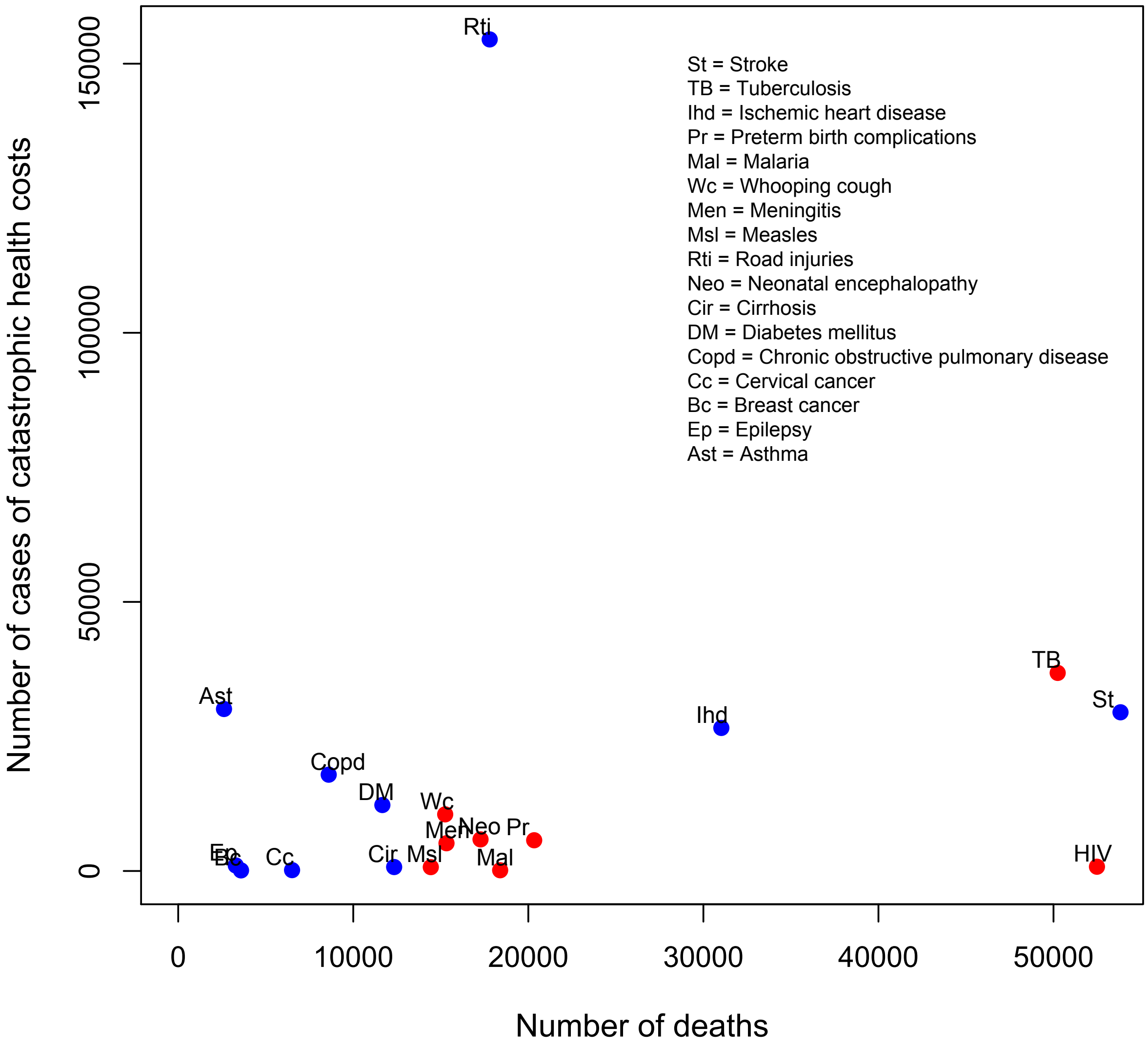

Note: Lri = lower respiratory infections, Dia = diarrhea, St = stroke, TB = tuberculosis, Ihd = ischemic heart disease, Pr = preterm birth complications, Mal = malaria, Rti = road traffic injuries, Neo = neonatal encephalopathy, Men = meningitis, Wc = whooping cough, Msl = measles, Cir = cirrhosis, DM = diabetes mellitus, Copd = chronic obstructive pulmonary disease, Cc = cervical cancer, Bc = breast cancer, Ep = epilepsy, Ast = asthma.

References

1. Ethiopia Central Statistical Agency and ICF International. 2011 Ethiopia Demographic and Health Survey: Key Findings. Calverton, Maryland, USA: CSA and ICF International, 2012.
2. Rudan I, Boschi-Pinto C, Biloglav Z, Mulholland K, Campbell H. Epidemiology and etiology of childhood pneumonia. Bulletin of the World Heath Organization 2008; 86:408-416.
3. Liu L, Johnson HL, Cousens S, Perin J, Scott S, et al. Global, regional, and national causes of child mortality: an updated systematic analysis for 2010 with time trends since 2000. Lancet 2012; 379:2151-61.
4. Memirie ST, Metafaria ZS, Norheim OF, Levin C, Verguet S, et al. Catastrophic health expenditure and impoverishment associated with pneumonia and diarrhea treatment costs in Ethiopia. Under review.
5. Lamberti LM, Fischer Walker CL, Black RE. Systematic review of diarrhea duration and severity on children and adults in low- and middle-income countries. BMC Public Health 2012; 12:276.
6. United Nations Population Division. World Population Prospects: the 2010 Revision. New York: United Nations, 2011.
7. Fischer Walker CL, Perin J, Aryee MJ, Boschi-Pinto C, Black RE. Diarrhea incidence in low- and middle-income countries in 1990 and 2010: a systematic review. BMC Public Health 2012; 12:220.
8. Jima D, Wondabeku M, Alemu A, Teferra A, Awel N, et al. Analysis of malaria surveillance data in Ethiopia: what can be learned from the Integrated Disease Surveillance and Response System? Malaria Journal 2012; 11:330.
9. World Health Organization. World malaria report 2014. Geneva: World Health Organization, 2014.
10. Deressa W, Hailemariam D, Ali A. Economic costs of epidemic malaria to households in rural Ethiopia. Tropical Medicine and International Health 2007; 12(10):1148-1156.
11. World Bank. World development indicators. Available from: http://data.worldbank.org/country/ethiopia (accessed August 5, 2015).
12. Kemp-Benedict E. Income distribution and poverty – Methods for using available data in global analysis. May 17, 2001. Available from: http://gdrs.sourceforge.net/docs/PoleStar_TechNote_4.pdf (accessed November 10, 2015).
13. Global health expenditure database. Geneva: World Health Organization, 2012. Available at: www.who.int/nha/country/en/index.html (accessed October 20, 2015)
14. World Health Organization. Global tuberculosis report 2014. Geneva: World Health Organization, 2014.
15. Datiko DG, Lindjørn B. Cost and cost-effectiveness of treating tuberculosis by health extension workers in Ethiopia: an ancillary cost-effectiveness analysis of community randomized trial. PLoS ONE 2010; 5(2):e9158.
16. Mudzi W, Stewart A, Musenge E. Case fatality of patients with stroke over a 12-month period post stroke. South African Medical Journal 2012; 102(9):765-767.
17. Maredza M, Bertram MY, Tollman SM. Disease burden of stroke in rural South

Africa: an estimate of incidence, mortality, and disability adjusted life years. BMC Neurology 2015; 15:54.

18. Zenebe G, Alemayehu M, Asmera J. Characteristics and outcomes of stroke at Tikur Anbessa teaching hospital, Ethiopia. Ethiopian Medical Journal 2005; 43(4):251-259.
19. Taddesse M, Norheim OF, Memirie ST, Verguet S, Johansson KA. Catastrophic health expenditure and impoverishment associated with cardiovascular disease treatment seeking in Ethiopia. Under review.
20. Central Statistical Agency [Ethiopia]. Ethiopia Mini Demographic and Health Survey 2014. Addis Ababa, Ethiopia, 2014.
21. Adam T, Lim SS, Mehta S, Bhutta ZA, Fogstad H, et al. Cost effectiveness analysis of strategies for maternal and neonatal health in developing countries. British Medical Journal 2005; 331:1107.
22. Beck S, Wojdyla D, Say L, Betran AP, Merialdi M, et al. The worldwide incidence of preterm birth: a systematic review of maternal mortality and morbidity. Bulletin of the World Health Organization 2010; 88:31-38.
23. Institute for Health Metrics and Evaluation. Global Burden of Disease Study 2013. Available from: http://vizhub.healthdata.org/gbd-compare/ (accessed May 25, 2015).
24. Gibbons L, Belizan JM, Lauer JA, Betrán AP, Merialdi M, Althabe F. The global numbers and costs of additionally needed and unnecessary caesarean sections performed per year: overuse as a barrier to universal coverage. World Health Report (2010). Background Paper, 30. Available from: http://www.who.int/healthsystems/topics/financing/healthreport/30Csectioncosts.pdf (accessed March 9, 2015).
25. UNAIDS. Global report: UNAIDS report on the global AIDS epidemic 2013. Geneva: UNAIDS, 2013.
26. Bikilla AD, Jerene D, Robberstad B, Lindjørn B. Cost-effectiveness of anti-retroviral therapy at a district hospital in southern Ethiopia. Cost Effectiveness and Resource Allocation 2009; 7:13.
27. Tagar E, Sundaram M, Condliffe K, Matatiyo B, Chimbwandira F, et al. Multi-Country Analysis of Treatment Costs for HIV/AIDS (MATCH): Facility-Level ART Unit Cost Analysis in Ethiopia, Malawi, Rwanda, South Africa and Zambia. PLoS ONE 2014; 9(11):e108304.
28. Rosen S, Long L. How much does it cost to provide antiretroviral therapy for HIV/AIDS in Africa? Health and Development Discussion Paper No.9. Center for International Health and Development Boston University School of Public Health: Boston, 2006.
29. Bhalla K, Harrison J, Shahraz S, Abraham JP, Bartels D, et al. Burden of Road Injuries in Sub-Saharan Africa. Department of Global Health and Population, Harvard School of Public Health, Boston, MA, africa.globalburdenofinjuries.org, 2013.
30. Kifle YA, Nigatu TH. Cost-effectiveness analysis of clinical specialist outreach as compared to referral system in Ethiopia: an economic evaluation. Cost Effectiveness and Resource Allocation 2010; 8:13.
31. Ramakrishnan M, Ulland AJ, Steinhardt LC, Moïsi JC, Were F, Levine OS. Sequelae due to bacterial meningitis among African children: a systematic literature review. BMC Medicine 2009; 7:47.
32. Stack ML, Ozawa S, Bishai DM, Mirelman A, Tam Y, et al. Estimated economic

benefits during the 'Decade of Vaccines' include treatment savings, gains in labor productivity. Health Affairs 2011; 30(6):1021-1028.

33. Eryd SA, Smith G, Melander O, Hedbad B, Engström G. Incidence of coronary events and case fatality rate in relation to blood lymphocyte and neutrophil counts. Arteriosclerosis, Thrombosis, and Vascular Biology 2012; 32(2):533-539.
34. Lang T, Ducimetière P, Arveiler D, Amouyel P, Cambou J-P. Incidence, case fatality, risk factors of acute coronary heart disease and occupational categories in men aged 30-59 in France. International Journal of Epidemiology 1997; 26(1):47-57.
35. Talley NJ, Locke R, Saito YA. GI Epidemiology. Oxford, UK: Blackwell Publishing, 2007.
36. Wolfson LJ, Grais RF, Luquero FJ, Birmingham ME, Strebel PM. Estimates of measles case fatality ratios: comprehensive review of community-based studies. International Journal of Epidemiology 2009; 38:192-205.
37. Présiozi M-P, Yam A, Wassilak SGF, Chabirand L, Simaga A, et al. Epidemiology of pertussis in a west African community before and after introduction of a widespread vaccination program. American Journal of Epidemiology 2002; 155:891-6.
38. Gizaw M, Harries AD, Ade S, Tayler-Smith K, Ali E, et al. Diabetes mellitus in Addis Ababa, Ethiopia: admissions, complications and outcomes in a large referral hospital. International Union Against Tuberculosis and Lung Disease 2015; 5(1):74-78.
39. Kirigia JM, Sambo HB, Sambo LG, Barry SP. Economic burden of diabetes mellitus in the WHO African region. BMC International Health and Human Rights 2009; 9:6.
40. Misganaw A, Hailemariam D, Ali A, Araya T. Epidemiology of major non-communicable diseases in Ethiopia: a systematic review. Journal of Health Population and Nutrition 2014; 32(1):1-13.
41. GLOBOCAN. Available from: http://globocan.iarc.fr/Pages/fact_sheets_population.aspx (accessed August 6, 2015).
42. Bjerkreim Strand K, Chisholm D, Fekadu A, Johansson KA. Scaling-up essential neuropsychiatric services in Ethiopia: a cost-effectiveness analysis. Health Policy and Planning 2015, 1–10 doi: 10.1093/heapol/czv093.
43. Ba-Diop A, Marin B, Druet-Cabanac, Nagoungou E, Newton CR, Preux P-M. Epidemiology, causes, and treatment of epilepsy in sub-Saharan Africa. Lancet Neurology 2014; 13:1029-44.
44. Woldeamanuel YW, Girma B, Teklu AM. Cancer in Ethiopia. Lancet 2013; 14:289-290.
45. Hailu A, Haile Mariam D. Patient side cost and its predictors for cervical cancer in Ethiopia: a cross sectional hospital based study. BMC Cancer 2013; 13:69.
46. To T, Stanojevic S, Moores G, Gershon AS, Bateman ED, et al. Global asthma prevalence in adults: findings from the cross-sectional world health survey. BMC Public Health 2012; 12:204.
47. Groot MT, Baltussen R, Uyl-de Groot CA, Anderson BO, Hortobágyi GN. Costs and health effects of breast cancer interventions in epidemiologically different regions of Africa, North America, and Asia. The Breast Journal 2006; 12(Suppl. 1):S81-S90.
48. Ethiopia Ministry of Finance and Economic Development. Ethiopia's progress towards eradicating poverty: an interim report on poverty analysis study (2010/2011).

March, 2012. www.mofed.gov.et/English/Resources/Documents/Interim%20Report%20 on%202010-11%20Poverty%20Analysis.pdf (accessed March 20, 2013).
49. Salem ABZ, Mount TD. A convenient descriptive model of income distribution: the gamma density. Econometrica 1974; 42(6):1115-27.
50. Kemp-Benedict E. Income distribution and poverty – Methods for using available data in global analysis. May 17, 2001. Available from: http://gdrs.sourceforge.net/docs/PoleStar_TechNote_4.pdf (accessed November 10, 2015).
